# Stress hormone levels in a freshwater turtle from sites differing in human activity

**DOI:** 10.1093/conphys/cow016

**Published:** 2016-05-17

**Authors:** Rebecca L. Polich

**Affiliations:** Department of Ecology, Evolution, and Organismal Biology, Iowa State University, Ames, IA 50011, USA

**Keywords:** Anthropogenic stressors, corticosterone, painted turtle, urban–rural gradient

## Abstract

Glucocorticoids, such as corticosterone (CORT), commonly serve as a measure of stress levels in vertebrate populations. These hormones have been implicated in regulation of feeding behaviour, locomotor activity, body mass, lipid metabolism and other crucial behaviours and physiological processes. Thus, understanding how glucocorticoids fluctuate seasonally and in response to specific stressors can yield insight into organismal health and the overall health of populations. I compared circulating CORT concentrations between two similar populations of painted turtle, *Chrysemys picta*, which differed primarily in the level of exposure to human recreational activities. I measured basal CORT concentrations as well as the CORT stress response and did not find any substantive difference between the two populations. This similarity may indicate that painted turtles are not stressed by the presence of humans during the nesting season. The results of this study contribute to our understanding of CORT concentrations in freshwater reptiles, a group that is historically under-represented in studies of circulating hormone concentrations; specifically, studies that seek to use circulating concentrations of stress hormones, such as CORT, as a measure of the effect of human activities on wild populations. They also give insight into how these species as a whole may respond to human recreational activities during crucial life-history stages, such as the nesting season. Although there was no discernable difference between circulating CORT concentrations between the urban and rural populations studied, I did find a significant difference in circulating CORT concentrations between male and female *C. picta.* This important finding provides better understanding of the sex differences between male and female painted turtles and adds to our understanding of this species and other species of freshwater turtle.

## Abstract (precis)

Although many turtle species are exposed to anthropogenic stressors, how these stressors impact circulating stress hormone levels in these species is widely unknown. I compared circulating levels of corticosterone in two populations of painted turtle differentially exposed to human recreation to assess the impact of anthropogenic stressors in this species.

## Introduction

Biologists have begun to recognize the importance of measuring the sublethal effects of anthropogenic stressors, and a growing literature exists that addresses this topic in birds (Strasser and Heath, 2013; Giraudeau and McGraw, 2014; Taylor *et al*., 2014), mammals (Pereira *et al*., 2006; Baria *et al*., 2007; Vick *et al*., 2012) and some reptiles (French *et al*., 2008, 2010). Many of these studies have demonstrated that, although certain species have adapted to human-modified landscapes, they often display altered stress hormone concentrations. Stress hormones, such as glucocorticoids, are involved in the regulation of a number of vital behaviours, such as locomotor activity and feeding, as well as metabolic processes, such as lipid metabolism. As such, they are believed to be crucial mediators of a suite of behaviours and physiological adaptations that can change with annual, expected events, such as breeding (O’Reilly and Wingfield, 2001) and migration (Ramenofsky and Wingfield, 2007), or acute, unpredictable stressors, such as El Niño (Steinfartz *et al*., 2007), acute predation (Monclús *et al*., 2008) and severe storms (Clutton-Brock, 1991).

Anthropogenic stressors can be considered chronic or acute depending on the regularity with which they occur. These human-induced stressors are particularly detrimental to wild vertebrates because it is unlikely that these organisms have evolved means to measure and mediate their effects via seasonal changes in physiology or via the glucocorticoid stress response. For example, human alterations to the environment could create situations where the glucocorticoid stress response is mismatched with the level of threat present. In this way, vertebrates may under-react to the threat, which could lead to death, or over-react to it, diverting resources to immediate survival and away from other life-history components, such as reproduction (Angelier and Wingfield, 2013). Such chronic over-reactions to stressors can have long-term fitness costs, such as decreased immune system functioning, suppression of growth, severe protein loss and inhibition of reproductive behaviour (Busch and Hayward, 2009). Thus, anthropogenic stressors can have especially detrimental effects on wildlife. For example, copperhead snakes (*Agkistrodon contortrix*) living in habitat cut by busy roads exhibit a reduced stress response in comparison to copperhead snakes living in habitat with lightly travelled roads (Owen *et al*., 2014). This result could indicate that prolonged chronic stress associated with living by busy roads has inhibited their ability to mount an adequate stress response. If so, then individuals in such circumstances might be more likely to be depredated or succumb to other perils, thereby decreasing their fitness.

Various studies also find direct evidence of human disturbances affecting important factors such as behaviour, immune function and fitness. For example, marine iguanas exhibit stress-induced decreases in immune function in populations exposed to anthropogenic disturbances (French *et al*., 2010). This observed reduction in immune function could induce population decline, because it is associated with increased parasite load and decreased ability to heal wounds. Likewise, elephants residing in areas of high poaching risk exhibited high faecal glucocorticoid concentrations and significantly lower reproductive output than elephants residing in areas of low poaching risk (Gobush *et al*., 2008). Thus, human stressors can affect wild populations at a number of different levels of biological organization. It is therefore crucial to appreciate how human disturbances influence wild populations so that we can gain a better understanding of future trajectories of these populations.

For this study, I examined the effect of human disturbance on stress hormone concentrations in a relatively unstudied taxon, turtles*.* Although endocrinological studies have been performed that examine circulating stress hormone concentrations (Cash *et al*., 1997; Selman *et al.*, 2012), this taxon is still under-represented compared with birds, mammals and other reptiles. I quantified circulating hormone concentrations in the painted turtle, *Chrysemys picta*, a small freshwater turtle that primarily occupies still bodies of water and can inhabit ponds in close proximity to human populations (Ernst and Lovich, 2009). I compared corticosterone (CORT) concentrations in two wild populations of *C. picta* that differ primarily in the amount of human recreational activity occurring in the summer when the turtles are most active. One population was exposed to minimal human disturbance, whereas the other was exposed to considerable human activity. I hypothesized that *C. picta* residing in environments that experience large amounts of human disturbance would exhibit higher baseline CORT concentrations or CORT stress responses than *C. picta* residing in environments that experience smaller amounts of human disturbance. Testing this hypothesis will add to our understanding of how freshwater turtles react to human recreational activities by yielding insight into how these activities affect circulating stress hormone concentrations. The nesting season, which occurs during part of the summer, is of particular importance to many freshwater turtle species because it a time of high fitness import for both male and female turtles. The nesting season is when eggs are laid; thus, production of offspring, a major component of organismal fitness, takes place during the nesting season. This is also when turtles are most likely to come into direct contact with humans (turtles bask frequently during the nesting season, and females must emerge onto the land to lay their eggs).

## Materials and methods

### Study populations

The study populations of *C. picta* are located ∼32 km apart in northwestern Illinois on the Mississippi River. The two populations differ primarily in the amount of human recreational activity to which they are exposed. The urban site, Thomson Causeway Recreational Area (TCRA; 41°57′N, 90°07′W; Schwanz *et al*., 2010), is a popular recreational vehicle campsite and daytime recreational use area. For example, in 2013 when this study was conducted, more than 7300 campers used this site between 31 March and 31 October (this number does not include the number of people who used this site as a daytime recreational area during that summer; personal communication from Kevin Zidarich, US Army Corps of Engineers). Nonetheless, *C. picta* frequently nest in close proximity to humans at this site (Bowen and Janzen, 2008; Strickland *et al*., 2010). The rural site, Lost Mound Unit (LMU; 42°27′N, 90°39′W), is an ex-military base. Military usage of this site peaked in 1945, with the number of personnel declining to 400 by 1995, 5 years before the site officially closed in 2000. Given that the munitions storage facilities are located upland and the base had few personnel present for many years, the turtle population in the river has not been exposed to sizeable amounts of human traffic for a considerable time.

In addition to the differences between these two sites in terms of terrestrial occupation by humans, they also differ substantially in aquatic usage by humans. For example, the portion of the Mississippi River backwaters flowing through LMU are off limits to human recreational activities because of the historical practice of firing munitions over the river while the site was a military base. In contrast, fishermen and other recreational boat users frequent the waters of TCRA throughout the summer. Given that daytime recreational use is not recorded at TCRA, exact numbers are not available for how many boats navigated the waters surrounding TCRA during the summers of 2012 and 2013. Although the two sites differ in aquatic and terrestrial usage by humans, they are fairly similar topographically. Thomson Causeway Recreational Area is located on an island in the Mississippi River close to mainland Illinois. The animals collected for this study were collected from a *C. picta* population that nests on the side of the island facing mainland Illinois; thus, these animals inhabit the backwaters of the Mississippi River. Likewise, the site at LMU was located on mainland Illinois, but was sheltered from the primary flow of the river by a series of islands close to the coast. Therefore, these turtles also inhabited the backwaters of the Mississippi River rather than the river itself. This is typical of *C. picta*, because they tend to prefer to live in ponds and backwaters rather than directly on major, fast-flowing rivers, such as the Mississippi.

Despite the differences between these two sites in terms of human recreational activities, the turtles inhabiting these sites are very similar in terms of genetic make-up. These two populations are part of a large clade within *C. picta* that spread into the central Great Plains and Rocky Mountain region after the retreat of the Laurentide ice sheets 20 000 years ago. This clade contains virtually no variation in mitochondrial DNA, indicating close genetic similarity between these two populations (Starkey *et al*., 2003).

### Fieldwork

Fieldwork was conducted over the course of 2 years; however, in the summer of 2012 the samples were collected only from TCRA. That summer, sample collection began on May 22 and ended on June 24. In the summer of 2013, fieldwork was conducted at both sites between 11 June and 20 July. Plasma samples for measurement of circulating CORT were obtained from turtles that had just finished nesting and from free-ranging male and female turtles captured while basking on logs. Basking turtles were captured using aquatic basking traps. These basking traps were constructed from four pieces of lumber (2″ × 4″) nailed together. A strip of fine mesh was attached to each board and formed a hoop at the bottom of the basking trap. This trap was then tied to an aquatic log on which painted turtles had previously been observed basking. Basking turtles were approached at a walk until they fled into the water in the middle of the basking trap. The turtle was then collected from the basking trap, and a blood sample was quickly taken. If it took >3 min to complete the approach and take the blood sample, that blood sample was not used in this study. These traps float at the surface of the water and capture turtles that flee into the water to escape perceived threats. At the urban field site, six males and one female were obtained through usage of the basking traps. At the rural site, 21 males and 11 females were obtained using basking traps.

I collected blood samples (0.25–0.5 ml) for baseline and stress-induced CORT analyses from the urban site in both 2012 and 2013 and from the rural site in 2013 (Table 1). I obtained basal blood samples within 3 min of capture from the caudal vein at the base of the tail using heparin-rinsed syringes and considered those samples to represent baseline concentrations (Refsnider *et al*., 2015). I separated plasma from red blood cells via centrifugation, snap-froze the plasma in liquid nitrogen and stored it at −80°C until analysis. I collected samples for stress-induced CORT analyses following a standard capture–restraint protocol similar to that used in many vertebrate studies to activate the adrenocortical axis (Aguirre *et al*., 1995; Cash *et al*., 1997; Wingfield and Ramenofsky, 1999; Jessop *et al*., 2004; Cash and Holberton, 2005; Palacios *et al*., 2012). Turtles were placed in plastic buckets, and additional blood samples were collected at 10, 30, 50, 90 and 180 min after time 0. I measured the body temperature after each blood sample was collected. All painted turtles used in this study, males and females from both sites, were confined in the same type of bucket and kept away from human activity in a shaded area. Thus, any stress associated with being confined would have been shared among all animals and would not have confounded the study. After blood collection was complete, I measured the plastron length for all turtles with callipers. To account for natural sources of variation, I sexed all turtles based on fore claw length, tail length and shape and overall body size; adult males have longer fore claws and more massive tails but smaller body sizes than adult females.

**Table 1:** Blood samples collected from wild *Chrysemys picta* for the years 2012 and 2013

Population	Year	Collection method: post-nesting/walking	Collection method: basking trap	Basal only	Stress response only	Total
Urban female	2012	8	0	0	8	8
Urban female	2013	30	1	23	8	31
Total urban female	2012 + 2013	38	1	23	16	39
Rural female	2013	0	11	8	3	11
Urban male	2012	3	0	0	3	3
Urban male	2013	1	6	4	3	7
Total urban male	2012 + 2013	4	6	4	6	10
Rural male	2013	0	21	13	8	21

Urban refers to the more human-impacted site, TCRA, whereas rural refers to the less human-impacted site, LMU. No blood samples were collected at LMU in 2012. Collection method, i.e. walking on land, post-nesting or basking trap (see *Materials and methods* section for details) is noted, as well as whether only a basal sample or an entire corticosterone stress-response series was collected.

### Corticosterone radioimmunoassay

I assayed total plasma CORT using a double-antibody radioimmunoassay (RIA) kit (catalogue no. 07-120103; MP Biomedical, Orangeburg, NY, USA) that has been validated in this study system (Refsnider *et al*., 2015). To assay total plasma CORT in *C. picta*, I used a modified version (Refsnider *et al*., 2015) of the MP Biomedical protocol for the ^125^I-CORT RIA. I diluted the *C. picta* samples 1:20 with steroid diluent, this dilution having been shown to stay best within the standard curve concentrations for *C. picta* (Refsnider *et al*., 2015). To validate the use of this RIA for *C. picta,* I tested for parallelism between the kit standards and the serial dilutions of a pool derived from my plasma samples (henceforth ‘plasma pool’, generated from 5 µl of plasma from five randomly chosen individuals from each year). The resulting curve was validated as parallel to the standard CORT curve after logit transformation (painted turtle, slope = −0.4473, *r*^2^* *= 0.9504; and CORT standards, −0.4621, *r*^2^*= *0.9834), confirming the validity of evaluation of circulating CORT in *C. picta* using this RIA (Robert *et al*., 2009). The pool and the low controls provided in the CORT RIA kit served as internal controls. For the CORT stress-response analysis, I analysed repeated samples for each time point in the stress-response series from a given individual in the same assay to minimize within-individual variation. Samples from different individuals from each population were randomly assigned to different assays using a random number generator (Palacios *et al*., 2012). In total, two assays were conducted. The first analysed the *C. picta* plasma samples collected in 2012, whereas the second analysed the *C. picta* plasma samples collected in 2013. The first assay contained 11 individuals and 66 samples (basal and stress-response series), and the second contained 43 individuals and 153 samples (basal and stress-response series). I calculated intra- and inter-assay coefficients of variation of percentage bound of the internal controls to assess assay precision. Average intra-assay variation was 7.19% and average inter-assay variation was 14.44%.

### Statistical analyses

I conducted all statistical analyses using the statistical program software SAS (SAS 9.3; SAS Institute Inc., Cary, NC, USA). For my analyses, I combined data from 2012 and 2013 at the urban field site to form a final CORT data set, because a preliminary analysis detected no statistical difference in CORT concentrations between these 2 years. Before analysis, I performed a Shapiro–Wilk test, which indicated that the CORT data set was not normally distributed. Therefore, I carried out log_10_ transformation of CORT concentrations to normalize the data. In addition, I removed one data point because it suggested a concentration of CORT higher than the highest concentration in the standard curve, and therefore could not be reliable, a known method of identifying outliers in hormone analyses (see Sparkman *et al*., 2014). Before conducting analysis of variance (ANOVA), I ised Akaike’s information criterion adjusted for finite sample sizes (AICc) as a model selection procedure to determine which independent variables I should include in my model. I conducted this analysis using the Proc GLMSELECT procedure in SAS, which performs effect selection in the framework of general linear models.

Based on model selection analysis, I evaluated baseline CORT using the following general linear model:

Y=μ+population+PL+sex+ϵ,

where *Y* is the dependent variable (also response variable), μ is the mean of the distribution of samples, and ϵ is an error term typically included in general linear models. It contains the variability of the dependent variable (*Y*) not explained by the independent variables. In addition, population is urban vs. rural, PL (plastron length) is a common proxy for size in turtles, and sex is male vs. female. Other independent variables included in the AICc model selection analysis included time of day (TOD), body temperature (BT), date (month, day, year), capture method (via aquatic basking log or post-nesting), assay (which RIA the sample was included in), as well as several interactions (PL × sex, date × sex, TOD × sex and population × sex). None of these variables was determined to be significant by the AICc model selection analysis, so I excluded them from the final model. I conducted the ANOVA procedure using the mixed-model procedure (Proc Mixed) in SAS software.

Likewise, I performed an AICc model selection analysis on my CORT stress-response data to determine which independent variables I should include in that model. Based on this analysis, I analysed stress-induced CORT using the repeated-measures general linear model:

Y=μ+population+PL+sex+time+date+ϵ.

As above, *Y* is the dependent variable (also response variable), μ is the mean of the distribution of samples, and ϵ is an error term typically included in general linear models. Additionally, population is urban vs. rural, PL is a common proxy for size in turtles, sex is the effect of male vs. female, time is the effect of the six repeated measures of CORT from the capture–restraint protocol, and date is when the sample was taken*.* Additional independent variables included in the AICc model selection analysis included time of day (TOD), body temperature (BT), method of capture and assay, as well as the interactions PL × sex, sex × time, sex × date, sex × TOD and sex × population. None of these variables was determined to be significant in the AICc model selection analysis, so I excluded them from the final model. After obtaining the model above, I conducted the ANOVA procedure using the mixed-model procedure (Proc Mixed) in SAS software.

In addition to the repeated-measures analysis for stress-induced CORT, I also performed statistical analyses using the area under the curve or integrated CORT response, the maximal CORT concentration achieved by each individual during the capture–restraint protocol, the time required by each individual to reach the maximal CORT concentration, and the amount of time required by each individual to descend to the basal concentration after reaching maximal CORT concentrations. However, none of these analyses yielded different insights from those already obtained. Thus, I exclude these results for simplicity.

## Results

Neither the baseline CORT analysis nor the CORT stress-response analysis indicated a major effect of the independent variable of interest, population (stress-response CORT, *r^2 ^*= 0.46, *F *= 0.41, *P = *0.53; and baseline CORT, *r*^2^ = 1, *F* = 0.00, *P* = 0.97). The variable, sex, was important in both cases. Male turtles exhibited overall higher CORT at all time points, including baseline, compared with female turtles. Male *C. picta* had almost double the mean basal concentrations of plasma CORT (±SEM), 35.77 ± 6.33 ng/ml, than did female *C. picta*, 19.13 ± 4.92 ng/ml (*F* = 8.68, *P* = 0.0049). For the capture–restraint protocol, male *C. picta* exhibited a higher overall stress response than did females (*F *= 12.58, *P *= 0.0009). For both basal CORT and stress-response CORT, there was a trend for larger individuals to exhibit lower basal and stress-response concentrations. However, in the basal analysis, PL fell short of being statistically significant (basal CORT, *F* = 3.08, *P* = 0.0857), whereas PL was statistically significant in the stress-response analysis (stress-response CORT, *F *= 4.03, *P* = 0.0463).

**Figure 1:**
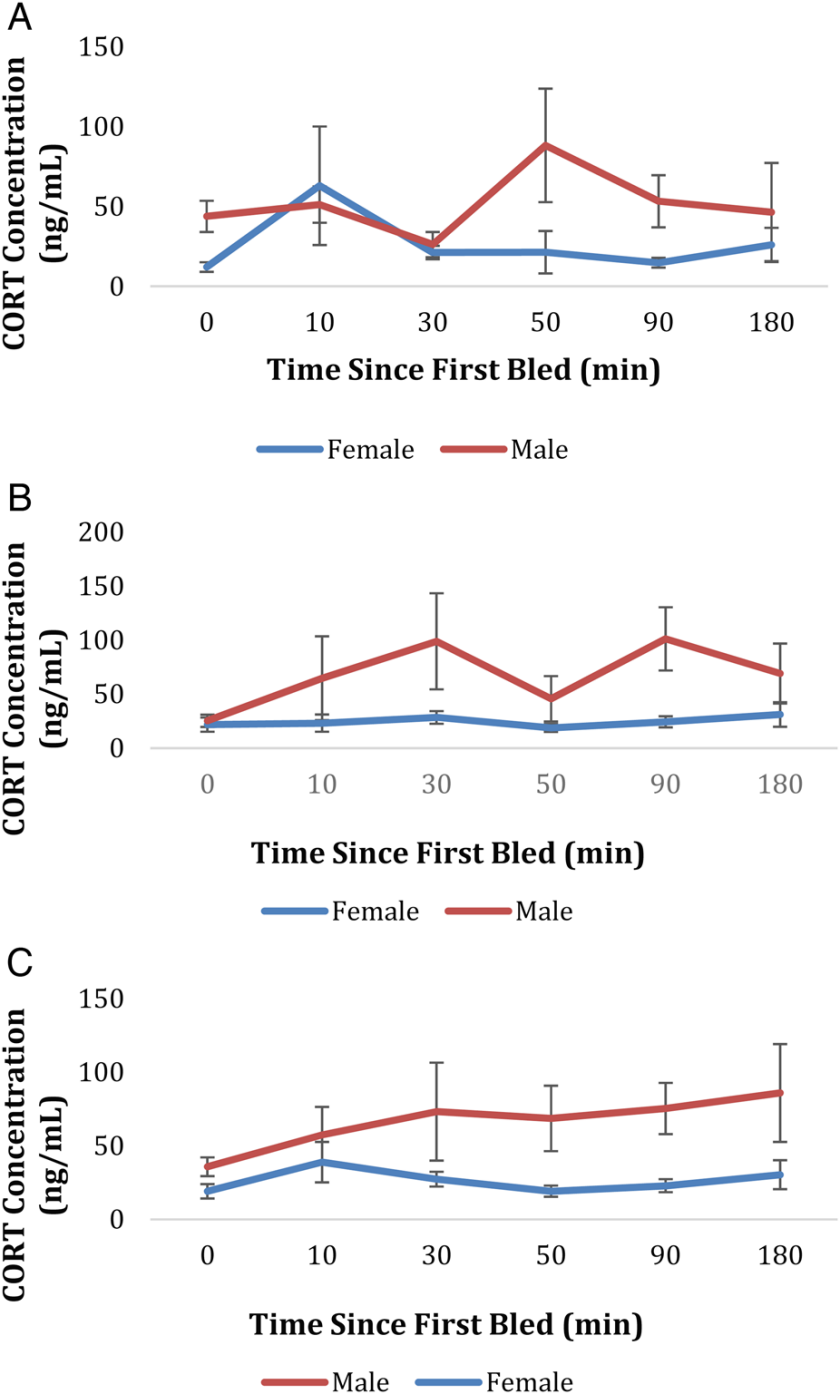
(**A**) the stress-response curve for *Chrysemys picta* at Lost Mound Unit (LMU), the less human-impacted population. Time = 0 min indicates baseline corticosterone concentrations (see *Materials and methods* section for details). The data are corticosterone concentration on the *y*-axis and time since first bled on the *x*-axis. Standard error bars have been inserted for each time point for each sex. Sample sizes basal: male, *n* = 13 and female, *n* = 8. Sample sizes stress response: male *n* = 8 and female *n* = 3. (**B**) The stress-response curve for *C. picta* at Thomson Causeway Recreational Area (TCRA), the more human-impacted population. Sample sizes basal: male, *n* = 4 and female, *n* = 23. Sample sizes stress response: male *n* = 6 and female *n* = 16. (**C**) This graph represents the *C. picta* stress-response curve from both the less human-impacted population, LMU, and the more human-impacted population, TCRA. Sample sizes basal only: male, *n* = 23 and female, *n* = 31. Sample sizes stress response: male *n* = 14 and female *n* = 19.

In the repeated-measures analysis, the time at which the blood sample was taken also affected the concentration of CORT measured. During the capture–restraint protocol, time point six (180 min) yielded the highest CORT concentration for males (85.87 ± 33.2 ng/ml) and time point two (10 min) produced the highest CORT concentration for females (38.87 ± 13.77 ng/ml; Fig. 1). However, the independent variable, date, did not affect CORT concentrations (*F* = 0.51, *P* = 0.47). The *r*^2^ value for both of these mixed models (baseline CORT and stress-response CORT) was calculated by adding up the total sum of squares for the model terms and then dividing that number by the total sum of squares. In the case of the baseline CORT model, the model terms used to calculate sum of squares were Julian date, PL, sex, population, individual identity and year. The total sum of squares was calculated by adding all of those terms as well as the residual. The final *r*^2^ value was 1. For the stress-response CORT model, the model terms used to calculate sum of squares were Julian date, PL, sex, population, time (which blood sample in the series the sample came from), individual identity and year. The total sum of squares was calculated by adding all of those terms as well as the residual. The final *r*^2^ value was 0.46.

## Discussion

As human populations continue to expand, wild populations must adapt to the new challenges they impose, or perish. Human encroachment does not necessarily result in complete loss of habitat. In some instances, such as National Parks, campsites and recreation areas, native habitat may remain largely unchanged. However, wild species inhabiting these areas must nevertheless adapt to the presence of often very large numbers of people throughout the year or during specific time periods. In cases where wild populations persist despite human-induced stressors, it is still often unclear how healthy these populations are compared with populations living in relatively undisturbed habitats.

I measured CORT concentrations in two wild populations of *C. picta*, one located at a highly human-impacted site and one located at a less human-impacted site, to gain insight into how freshwater turtles are affected by human recreational activities. I found no evidence that *C. picta* residing in highly human-impacted habitats exhibit higher stress levels than their counterparts living in less human-impacted areas. Interestingly, in a concurrent behavioural study performed by myself and M. Barazowski on these same populations, we did find evidence that *C. picta* are able to modify flight initiation distance behaviour based on levels of human recreational activity. The results of that study suggest that *C. picta* are able to habituate to the physical presence of relatively large numbers of humans (Polich RL and Barazowski M, unpublished observations).

I found no evidence to support the hypothesis that *C. picta* exposed to humans during the nesting season would exhibit heightened CORT concentrations, either baseline or stress induced. If circulating CORT concentrations are a proxy for stress, this finding suggests that *C. picta* are not stressed by the presence of humans during the nesting season. Heightened CORT at the urban site would have indicated that, compared with the rural population, the urban population is more stressed. As the urban and rural sites were ∼32 km apart on a similar stretch of the Mississippi River and *C. picta* at these sites exhibit virtually no genetic variation between one another (Starkey *et al*., 2003), this similarity in CORT concentrations implies either that the urban population has habituated to the presence of humans or that *C. picta* are not stressed by the presence of humans regardless of whether they have been exposed to them previously. This finding is in contrast to previous studies of songbirds and snakes (Moore *et al*., 2001; Deviche *et al*., 2014; Lattin *et al*., 2014). The results from these previous studies may differ from my own because *C. picta* are long lived, whereas most of the previously studied taxa are not. Evolutionary theory and some previous research has demonstrated that physiological as well as behavioural plasticity may help long-lived organisms, such as *C. picta*, to survive dramatic environmental shifts (Levins, 1968; West-Eberhard, 1989; Dickinson *et al*., 1991; Bruno and Edmunds, 1997). This would be advantageous to long-lived organisms because they have long generation times and may not respond to environmental perturbations rapidly enough if they rely on evolution alone. It is also possible that *C. picta* is a remarkably plastic species even for a long-lived vertebrate, and this plasticity has contributed to its incredible success as a species. For example, *C. picta* are plastic in nest-site choice behaviour, resulting in similar incubation regimes and nest sex ratios across the vastly different climates that this species inhabits (Refsnider and Janzen, 2012). This ability to modify behaviour and physiology in response to a variable climate has probably led to the success of *C. picta* as a wide-ranging species and may help it adapt to climate-warming scenarios (McGaugh *et al*., 2010). It is also possible that *C. picta* simply are not stressed by the presence of humans. Thus, even a naïve population of *C. picta* that has never been exposed to human disturbances would not exhibit different concentrations of circulating CORT compared with a population that has never been exposed to human disturbance.

It is possible that a stress difference does exist between these populations and that my methods were unable to detect it. For example, I collected more samples from females than males at the urban site (total females = 39, total males = 10) and the opposite at the rural site (total females = 11, total males = 21). In order to gain some insight into my ability to detect a biologically relevant sample effect with the sample sizes that I collected, I conducted a *post hoc* power analysis of the basal and the CORT stress-response data sets. These analyses revealed a power of 0.473 for both basal CORT and the CORT stress response. Thus, power I observed for this study is lower than the standard power accepted for adequacy, 0.8. However, *post hoc* analyses of power are not widely accepted among biologists or statisticians, particularly for non-significant results. This is because power is directly related to the *P*-value of the statistical test performed. Thus, when a *P*-value is not significant (as in the present study), power will necessarily be low. Indeed, in nearly all cases when a *P*-value is >0.05, the *post hoc* power will be 0.5 or lower (Goodman and Berline, 1994; Hoenig and Heisey, 2001; Brosi and Biber, 2009). Therefore, although the *post hoc* power for this analysis is low, it is unlikely that it invalidates the results reported in this manuscript.

In addition, having fewer *C. picta* present at the rural site, and capturing more of them later in the season, could have biased the results. Intra-annual variation in CORT concentrations has been documented in other species and has been attributed to changes in behaviour or to interactions between CORT and sex hormones, such as testosterone. For example, baseline concentrations of CORT in female green sea turtles (*Chelonia mydas*) increase during the nesting season (Hamann *et al*., 2002). In another study, Selman *et al*. (2012) found that basal concentrations in the map turtle (*Graptemys flavimaculata*) stayed largely the same for both females and males. However, females exhibited a dampened CORT stress response during the nesting season, whereas males exhibited a heightened stress response during the nesting season. Furthermore, previous studies in other vertebrates and in some reptiles have shown a dampened stress response in all females during the nesting season (Astheimer *et al*., 1995; Breuner *et al*., 2003) or specific to gravid females during the same period (Jessop *et al*., 2000; Anderson *et al*., 2014).

Nevertheless, for the population and the species studied here, such a bias may be unlikely because the variable, date, did not have a significant effect on CORT. This may be the case because all samples were collected during the nesting season. In fact, some of the last blood samples collected at my more human-impacted site came from female turtles who had just completed nesting, and gravid females were collected from my less human-impacted site until the day that collection ceased. Furthermore, *C. picta* females exhibit minimal intra-annual variation in CORT concentrations (Refsnider *et al*., 2015). That study compared CORT concentrations at four time periods throughout the year in four populations from across the *C. picta* range (TCRA, Washington, Iowa and New Mexico). Baseline concentrations of CORT did not vary significantly for any population or time period except for immediately after emergence from hibernation (Refsnider *et al*., 2015). Nonetheless, it would be useful to assess the generality of my findings by including more paired urban and rural sites and by studying freshwater turtle species that are habitat specialists as opposed to generalists, such as *C. picta*. It would also be useful to assess heterophil-to-lymphocyte ratios. After exposure to a stressor, heterophil-to-lymphocyte ratios remain elevated long after CORT concentrations have declined, suggesting that heterophil-to-lymphocyte ratios may be a more reliable measure of long-term stress than circulating CORT (Vleck *et al*., 2000; Davis *et al*., 2008). Nonetheless, given the abundance of studies that have successfully shown CORT to be an indicator of stress induced by human activity, the CORT data collected here should be sufficient to determine whether *C. picta* are stressed by the presence of humans or not.

One intriguing finding from the present study is that male *C. picta* have significantly higher circulating CORT concentrations than female *C. picta*. Females may have depressed concentrations of CORT compared with males because the stressors associated with the nesting season, such as coming onto land to search for suitable nesting habitat (females may be depredated, or injured or killed by automobiles), are more substantive for females than they are for males. For example, some bird species have depressed CORT concentrations during the reproductive season because high CORT concentrations are associated with self-maintenance behaviours as opposed to breeding behaviours (Breuner *et al*., 2003; Palacios *et al*., 2012). It could be that female *C. picta* have depressed basal CORT and a depressed CORT stress response for the same reason. However, this explanation may be unlikely, because previous research detected no intra-annual fluctuation of CORT concentrations in female *C. picta* (Refsnider *et al*., 2015). In species that exhibit depressed CORT concentrations attributable to nesting pressures, typically it is only during the nesting season itself that CORT concentrations are lowered, as opposed to them being consistently lower (Breuner *et al*., 2003). Alternatively, male-specific stresses, such as searching for females and convincing them to mate, are more apt to elicit a heightened stress response in *C. picta*. Indeed, CORT has been associated with increases in locomotor activity because of breeding season activities in amphibians and other reptiles (Landys *et al*., 2006). To determine the validity of this hypothesis, it would be useful to obtain intra-annual blood samples from male *C. picta* to determine whether the stress response is heightened only during the breeding season.

An additional notable finding is that *C. picta* appear to have higher concentrations of CORT compared with other species of turtle. For example, Gregory and Schmid (2001) found CORT baselines of 6.16 ± 2.31 ng/ml in Kemp’s ridley sea turtles, roughly one-half to one-quarter the concentrations reported here. However, the *C. picta* results were consistent across 2 years of data collection, and previous work on female *C. picta* found similar high concentrations of basal CORT (Refsnider *et al*., 2015). Therefore, these high basal concentrations are likely to reflect genuine circulating concentrations of CORT within wild *C. picta.* Furthermore, some other species of wild turtle have been shown to have higher concentrations of circulating CORT than those reported by Gregory and Schmid (2001); Drake *et al*. (2012) reported basal CORT concentrations of ∼8 to ∼9 ng/ml. In addition, although the CORT stress-response concentrations recorded for *C. picta* are higher than those documented in other species of turtle, many of these other studies took only a basal and a ‘time 30’ blood sample. They therefore may not have captured the entire stress response (Cash *et al*., 1997; Gregory and Schmid, 2001; Selman *et al*., 2012).

The results of the present study indicate that *C. picta* does not experience heightened CORT concentrations in response to the extensive presence of humans during the nesting season. However, males exhibited an elevated stress response compared with females. Future studies in this and other systems should evaluate these findings for generality in freshwater turtles as a whole. Regardless, this study contributes basic data on glucocorticoid responses for a free-living, freshwater reptile, a group that is historically under-represented in glucocorticoid studies. This information is essential for building a comprehensive data set that allows the testing of comparative endocrinological and physiological hypotheses across reptiles. Given that reptiles are the only ectothermic amniotes and occupy a key phylogenetic position within vertebrates, insight into their endocrinology may also help to answer important questions about the evolution of this physiological system.

## Funding

This work was supported by a grant from the National Science Foundation to F. Janzen (DEB-1242510).
